# Antimicrobial Behavior of Surface-Treated Commercially Pure Titanium (CpTi) for Dental Implants in Artificial Saliva—In Vitro Study

**DOI:** 10.3390/antibiotics14070715

**Published:** 2025-07-16

**Authors:** Roshni Bopanna, Neetha J. Shetty, Ashith M. Varadaraj, Himani Kotian, Sameep Shetty, Simran Genescia

**Affiliations:** 1 Department of Periodontology, Manipal College of Dental Science Mangalore, Manipal Academy of Higher Education, Manipal 576 104, Karnataka, India; roshni.k1@learner.manipal.edu (R.B.); simran.mcodsmlr2022@learner.manipal.edu (S.G.); 2 Department of Orthodontics and Dentofacial Orthopaedics, Manipal College of Dental Science Mangalore, Manipal Academy of Higher Education, Manipal 576 104, Karnataka, India; ashith.mv@manipal.edu; 3 Lecturer cum Biostatistician, Department of Community Medicine, Kasturba Medical College Mangalore, Manipal Academy of Higher Education, Manipal 576 104, Karnataka, India; himani.kotian@manipal.edu; 4 Department of Oral and Maxillofacial Surgery, Manipal College of Dental Science Mangalore, Manipal Academy of Higher Education, Manipal 576 104, Karnataka, India; sameep.shetty@manipal.edu

**Keywords:** dental implants, CpTi, surface coatings, antimicrobial activity, doped hydroxyapatite

## Abstract

**Background/Objectives**:Titanium implant surface modifications enhance osseointegration and prevent microbial colonization, improving implant longevity. Antimicrobial coatings, particularly cerium- and bismuth-doped hydroxyapatite (CeHAp and BiHAp), have gained attention for reducing infection-related complications. This study evaluates the antimicrobial activity of CeHAp and BiHAp coatings on CpTi compared to untreated CpTi in artificial saliva at pH levels of 4.5, 6.5, and 8. **Methods**: Antibacterial efficacy against *Staphylococcus aureus* (*S. aureus*), *Escherichia coli* (*E. coli*), and *Candida albicans* (*C. albicans*) was assessed using the broth dilution method. Titanium rods coated with test compounds were incubated in inoculated nutrient broth, and microbial inhibition was determined via optical density at 600 nm. A statistical analysis was performed using the Kruskal–Wallis ANOVA test, the median and Interquartile Range were determined for the variables, and a Dwass–Steel–Critchlow–Fligner intergroup pairwise comparison was conducted. **Results**: The results showed that both the CeHAp and BiHAp coatings demonstrated significant antimicrobial activity against *S. aureus* (OD = 0.01) at pH 6.5, which was more pronounced than the activity observed against *E. coli* (OD = 0.05), with the difference being statistically significant (*p* = 0.001). The least antimicrobial activity was observed against *C. albicans* (0.21) at pH 8 (*p* = 0.001). **Conclusion**: These findings highlight the pH-dependent effectiveness of BiHAp and CeHAp coatings in inhibiting microbial growth. Their application on titanium implants may enhance antimicrobial properties, contributing to improved dental implant success and broader biomedical applications.

## 1. Introduction

The exceptional properties of CpTi and its alloys, including biocompatibility, corrosion resistance, and favorable mechanical characteristics, have established them as widely used biomaterials in dental implantation [1,2]. While osseointegration is crucial, the health of the surrounding soft tissue also determines long-term implant success [3]. However, biofilm formation on implant surfaces poses a major threat to clinical outcomes [4,5]. The rapid colonization of titanium by microbes such as *S. aureus*, *E. coli*, and *C. albicans*—common in peri-implantitis—underscores the need for antimicrobial interventions [6,7,8].

Surface modification strategies using antibacterial coatings have shown promise in mitigating infection risk [9,10]. Among these, cerium- and bismuth-doped hydroxyapatite (CeHAp and BiHAp) exhibit broad-spectrum antimicrobial properties, affecting cell membrane integrity and microbial viability [11,12]. While previous studies have demonstrated their efficacy, few have evaluated their performance across different pH conditions that mimic the dynamic oral environment [13,14,15,16].

This in vitro study was, therefore, planned to compare and evaluate the antimicrobial properties of CpTi without a surface coating compared to CpTi coated with CeHAp and BiHAp in the presence of inoculated microorganisms—*S. aureus*, *C. albicans*, and *E. coli*—in artificial saliva at different pHs—4.5, 6.5, and 8.

## 2. Results

Data was summarized as the median and Interquartile Range. The results obtained were subjected to statistical analysis for an assessment and comparison of the two antimicrobial surface coatings on CpTi with varying salivary pHs of artificial saliva. The antimicrobial coating on CpTi did not support a normal distribution as per the Kolmogorov–Smirnov test. A statistical analysis was performed using the non-parametric Kruskal–Wallis ANOVA test, the median and Interquartile Range were determined for the variables, and a Dwass–Steel–Critchlow–Fligner intergroup pairwise comparison was conducted using commercially available software SPSS 20.0 (IBM, Chicago, IL, USA). A *p* value < 0.05 was considered statistically significant.

A comparative analysis of the microbial growth of *S. aureus*, *E. coli*, and *C. albicans* on the three samples—CpTi, BiHAp, and CeHAp—at salivary pH levels of 4.5, 6.5, and 8 was carried out with a Kruskal–Wallis ANOVA, and a *p* value < 0.05 was considered statistically significant.

*S. aureus* at pH 4.5 in the CpTi group exhibits the highest median value (0.1), while the BiHAp group demonstrates the lowest median value (0.003), indicating that there is no statistically significant difference among the three groups.

*E. coli* at pH 4.5 in the CeHAp group presents the highest median value (0.18), with the BiHAp group exhibiting the lowest (0.05), indicating that there is no statistically significant difference among the three groups.

*C. albicans* at pH 4.5 in the CpTi group records the highest median value (0.14), while the BiHAp group has the lowest (0.04), displaying a statistically insignificant difference (Table 1).

*S. aureus* at pH 6.5 in the CpTi group displays the highest median value (0.24), while the BiHAp group exhibits the lowest (0.0067), showing a statistically significant difference (*p* = 0.025) among the three groups.

*E. coli* at pH 6.5 in the CpTi group displays the highest median value (0.27), while the CeHAp group exhibits the lowest (0.05), showing statistical significance (*p* = 0.027).

*C. albicans* at pH 6.5 in the CpTi group displays the highest median value (0.16), while the BiHAp and CeHAp groups exhibit the same value (0.1), showing a statistically insignificant difference (Table 2).

The Dwass–Steel–Critchlow–Fligner pairwise comparison procedure was used to determine any significant differences between pairs of groups. *S. aureus* at pH 6.5 was statistically significant according to the Kruskal–Wallis test (*p* value = 0.025). The Dwass–Steel–Critchlow–Fligner pairwise comparison (non-parametric test) revealed that for CpTi vs. BiHAp, all the pairwise differences showed statistically insignificant results (Table 3).

*E. coli* at pH 6.5 showed statistically significant differences according to the Kruskal–Wallis test (*p* value = 0.027). The Dwass–Steel–Critchlow–Fligner pairwise comparison (non-parametric test) revealed that for CpTi vs. BiHAp, all the pairwise differences showed statistically insignificant results (Table 4).

*C. albicans* at pH 6.5 in the CpTi group displays the highest median value (0.16), while the BiHAp and CeHAp groups exhibit the same value (0.1), showing statistical insignificance (Table 2).

*S. aureus* at pH 8 in the BiHAp group displays the highest median value (0.12), while the CpTi group exhibits the lowest (0.2), showing a statistically significant difference (*p* = 0.033).

*E. coli* at pH 8 in the BiHAp group displays the highest median value (0.15), while the CpTi group exhibits the lowest (0.1), showing a statistically significant difference (*p* = 0.044).

*C. albicans* at pH 8 in the BiHAp group displays the highest median value (0.16), while the CeHAp group exhibits the lowest (0.2), showing a statistically significant difference (*p* = 0.048) (Table 5).

*S. aureus* at pH 8 showed statistically significant differences according to the Kruskal–Wallis test (*p* value = 0.033). The Dwass–Steel–Critchlow–Fligner pairwise comparison (non-parametric test) revealed that for CpTi vs. BiHAp, all the pairwise differences showed statistically significant results (Table 6).

*E. coli* at pH 8 showed statistically significant differences according to the Kruskal–Wallis test (*p* value = 0.044). The Dwass–Steel–Critchlow–Fligner pairwise comparison (non-parametric test) revealed that for CpTi vs. BiHAp, all the pairwise differences showed statistically significant results (Table 7).

*S. aureus* at pH 8 shows statistical significance (*p* value = 0.028). Hence a Dwass–Steel–Critchlow–Fligner pairwise comparison (non-parametric test) was performed, which reveals that the control (W = 1.59, *p* value 0.5), BiHAp group (W = 2.78; *p* value = 0.121), CeHAp group (W = 2.78; *p* value = 0.121), and all the combinations showed statistical significance (Table 8).

## 3. Discussion

Titanium and its alloys are favored in dental implantology for their mechanical and biological compatibility [1,2]. However, their lack of inherent antimicrobial activity makes them susceptible to early biofilm formation and microbial colonization, which are key contributors to peri-implant disease and implant failure [6,8]. Our study evaluated whether the surface coatings of CeHAp and BiHAp on CpTi could mitigate this microbial risk under varying pH conditions that simulate dynamic intraoral environments [12,17].

One of the key findings was the pronounced reduction in microbial growth at pH 6.5, particularly for *S. aureus*, a keystone pathogen in peri-implantitis. CeHAp coatings at this pH exhibited significantly lower optical density readings, suggesting superior antimicrobial performance [11,18]. This is of clinical interest because pH 6.5 reflects early post-operative saliva conditions, during which microbial suppression can critically influence the trajectory of healing and implant integration [19].

The pH-dependent nature of antimicrobial efficacy represents a biologically relevant insight [20]. While earlier studies evaluated coatings under static, often neutral pH conditions, we introduced acidic and alkaline challenges that mimic those encountered in real oral environments due to diet, inflammation, or oral hygiene practices [21,22]. This adds translational value to our findings and reveals that CeHAp coatings retain antimicrobial behavior across pH ranges, although with optimal performance at neutral pH [23].

Interestingly, while both CeHAp and BiHAp demonstrated antimicrobial properties, CeHAp consistently outperformed BiHAp against all test organisms [24,25]. This could be attributed to cerium’s redox-active nature, which disrupts microbial oxidative balance and may interfere with DNA and protein synthesis [11,26]. However, despite this advantage, neither coating showed strong efficacy against *C. albicans*, indicating a selective spectrum of action that may be insufficient for fungal colonizers without additional modifications [27,28].

Critically, the antimicrobial effect, although statistically significant in some comparisons, was modest in magnitude, particularly when viewed through intergroup pairwise analyses. Moreover, the small sample size per group (n = 3) reduces the power of these comparisons and may limit the reproducibility of the findings. The reliance on optical density alone as a surrogate for microbial inhibition, without accompanying colony-forming unit (CFU) counts or microscopy, also restricts mechanistic understanding [29].

Importantly, our model used aerobic microorganisms, which, although relevant for early colonization, do not represent the anaerobic polymicrobial biofilms implicated in chronic peri-implantitis [30,31]. This disconnect limits the ecological validity of this study. Moreover, we did not assess host cell interactions, such as osteoblast viability or immune cell response, which are essential to substantiate claims of improved implant compatibility or osseointegration [32].

Nevertheless, this study represents a meaningful step toward pH-aware biomaterial testing, an area underexplored in the current literature. The observed antimicrobial trends, especially the superiority of CeHAp at physiologic pH, support the further development of this coating as a preventive strategy against early-stage peri-implant microbial adhesion [11,13,33]. Future research should employ anaerobic biofilm models, co-culture systems, and long-term in vivo studies to bridge the gap between in vitro efficacy and clinical application.

In conclusion, our findings suggest that CeHAp-coated CpTi implants may offer adaptive antimicrobial protection in the early stages of implant healing, particularly under neutral pH conditions. However, further mechanistic and translational studies are necessary to evaluate their long-term biological performance and clinical relevance [11,23,33,34].

## 4. Materials and Methods

### 4.1. Study Design and Number of Participants

The objective of this in vitro study was to assess the antimicrobial effectiveness of two surface coating types applied to CpTi implants: CeHAp and BiHAp. This study included 45 titanium rod specimens, which were divided into three groups: CeHAp-coated CpTi, BiHAp-coated CpTi, and uncoated CpTi (control). With five replicates per condition, each group was further separated according to exposure to three distinct pH environments (4.5, 6.5, and 8).

Based on antimicrobial efficacy data from a previous study by Ciobanu et al. [31], considering the highest antimicrobial standard deviation of 10 and assuming an α error of 5% and a power of 80%, a total of 45 samples were included in this study using the following formula:

$$N=\frac{{2\left(Z_{1}-\frac{\alpha }{2*k}+Z_{1}-\beta \right)}^{2}\sigma^{2}}{d^{2}}$$

where Z (1 − α/2∗k) = the Z score for the α error chosen.

Z (1 − β) = the Z score for the power chosen.

σ = the average standard deviation.

d = the minimum difference between values that make a clinically relevant impact = 7.21 (clinically significant difference).

The primary endpoint is the antimicrobial activity of pure Ti (quantitative outcome).

### 4.2. Preparation of Titanium Substrate

**Figure 1:**
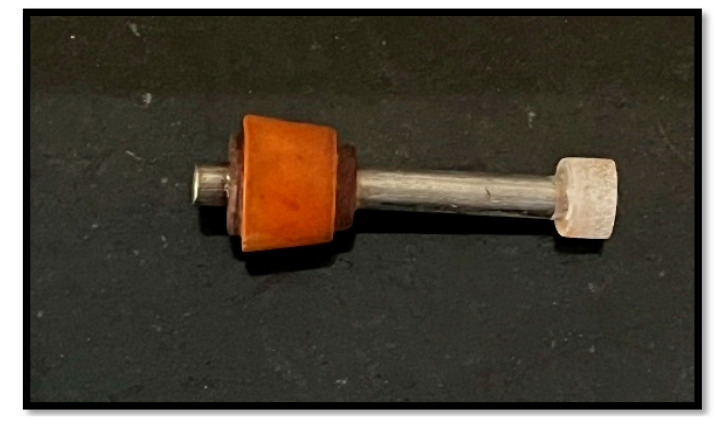
CpTi rod used in this study.

In order to create cylindrical specimens that were 40 mm in length and 8 mm in diameter, CpTi rods were procured from Mishra Dhatu Nigam Limited, Hyderabad, India. Using cold-cure epoxy resin (Amkay′s Epoxy Resin by Amkay Chem, Rajasthan, India), each rod was mounted to reveal a single flat surface that measured roughly 0.95 cm^2^ (Figure 1). This surface was intended for microbiological exposure and antimicrobial coating.

**Figure 2:**
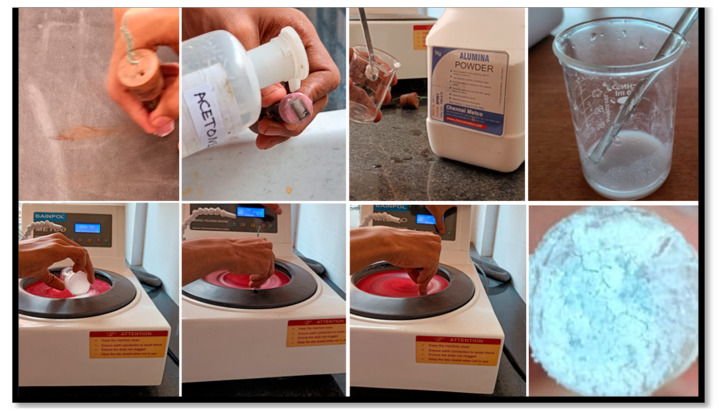
Surface preparation of CpTi.

Standard metallographic processes were used to polish the exposed titanium surfaces. To eliminate surface imperfections, belt grinders (80 micron) and progressively finer grades of emery paper were used for 20 min for the initial polishing. A mirror-like finish was then achieved by a final polishing with levigated alumina on a polishing wheel. In order to prepare the specimens for coating, they were polished, then ultrasonically cleaned in acetone to eliminate any organic impurities, rinsed well with double-distilled water, and dried in a hot air oven at 37 °C (Figure 2) [23].

### 4.3. Preparation of Antimicrobial Coatings

#### 4.3.1. Cerium-Doped Hydroxyapatite (CeHAp) Coating

A highly concentrated calcification solution (SCS) was created by dissolving calcium chloride dihydrate (CaCl_2_·2H_2_O), sodium dihydrogen phosphate monohydrate (NaH_2_PO_4_·H_2_O), and sodium bicarbonate (NaHCO_3_) procured from Medi Biotronics, Bangalore, India, in 1 L of deionized water with vigorous stirring. The SCS had specific ion concentrations of 6.5 mmol/L Na^+^, 10 mmol/L Ca^2+^, 20 mmol/L Cl^−^, 5 mmol/L H_2_PO_4_^−^, and 1.5 mmol/L HCO_3_^−^, maintaining a Ca/P atomic ratio of 1.67. A modified version of the highly concentrated calcification solution (Ce-SCS) was prepared by incorporating a suitable amount of cerium (IV) sulphate tetrahydrate (Ce[SO_4_]_2_·4H_2_O) into the original SCS while ensuring the (Ce + Ca)/P and xCe ratios remain at 1.677 and 0.005, respectively. The concentration of cerium used was around 0.5%, falling within the therapeutic range. To treat titanium samples, they were immersed directly into 200 mL of Ce-SCS contained in a glass beaker, maintained at 37 °C in a shaking water bath. Ce-SCS was refreshed every 12 h to maintain stable ion concentrations. After specific immersion periods (24 h), the titanium rod was removed from the solutions, rinsed with deionized water, and dried in an oven at 37 °C for 1 h [26].

#### 4.3.2. Bismuth-Doped Hydroxyapatite (BiHAp) Coating

The same method as above was used to create a supersaturated calcification solution (SCS). This solution contained ion concentrations of 6.5 mmol/L Na^+^, 10 mmol/L Ca^2+^, 20 mmol/L Cl^−^, 5 mmol/L H_2_PO_4_^−^, and 1.5 mmol/L HCO_3_^−^, maintaining a calcium-to-phosphorus (Ca/P) atomic ratio of 1.67, resembling biological hydroxyapatite. Upon the addition of bismuth nitrate pentahydrate (Bi (NO_3_)_3_∙5H_2_O) salt to the original SCS, a modified SCS (Bi-SCS) was obtained. In this modified solution, the atomic ratios of (Bi + Ca)/P and xBi = Bi/(Bi + Ca) were 1.67 and 0.01, respectively. The concentration of bismuth in Bi-SCS is approximately 1%.

#### 4.3.3. Spin Coating Method

Both the CeHAp and BiHAp coatings were deposited using the spin coating technique, a well-established method in thin film technology. This technique utilizes centrifugal forces to uniformly coat a substrate. This process enables the uniform dispersion of the coating material on the titanium substrate while also allowing for control over the thickness of the coated layer. Using centrifugal force, a small amount of coating solution is applied to the polished surface, and the sample is spun rapidly (usually between 3000 and 5000 rpm) to distribute the coating evenly. Following application, the coated specimens were dried at 37 °C to harden the coatings.

### 4.4. Preparation of Artificial Saliva

Using the Fusayama–Meyer formula, artificial saliva was prepared to mimic the ionic environment that exists inside the oral cavity. Disodium hydrogen phosphate (0.58 g), potassium chloride (0.4 g), sodium chloride (0.4 g), calcium chloride (0.6 g), and urea (1.0 g) were all dissolved in one liter of distilled water to form the base solution [34].

In order to mimic the different pH levels normally seen in the oral cavity, the following were used:

- For pH 4.5: A total of 1.5 mL of glacial acetic acid, 100 mL distilled water, and 0.64 gm of sodium acetate were used to form an acetic acid–sodium acetate buffer of pH 4.5.
- For pH 6.5: A total of 2.85 gm of diethyl barbituric acid and 14.2 gm of sodium diethyl barbititrate were dissolved in 1 L distilled water to form a barbitone buffer of pH 6.5.
- For pH 8: A total of 2.12 gm of anhydrous sodium carbonate and 1.68 gm of sodium bicarbonate were dissolved in 100 mL distilled water to form a carbonate–bicarbonate buffer of pH 8.

Each titanium rod was then subjected to one of these three pH conditions.

### 4.5. Culture Media Used

- Media A (control): Containing CpTi without surface treatment in artificial saliva at pHs 4.5, 6.5, and 8.
- Media B (test): Containing CpTi coated with CeHAp in artificial saliva and at pHs 4.5, 6.5, and 8.
- Media C (test): Containing CpTi coated with bismuth-doped hydroxyapatite in artificial saliva at pHs 4.5, 6.5, and 8.

### 4.6. Microorganisms and Inoculum Standardization

Three prevalent microorganisms, including bacterial and fungal pathogens, that are frequently linked to peri-implant diseases were used in this study. These included the Gram-positive cocci *S. aureus*, the Gram-negative bacilli *E. coli*, and the pathogenic yeast *C. albicans*. While *C. albicans* was isolated from a clinical specimen and cultivated in nutrient broth, *S. aureus* and *E. coli* were acquired as standard microbiological strains from the Microbial Type Culture Collection (MTCC), Chandigarh, India.

A 0.5 McFarland standard, which translates to an approximate bacterial concentration of 1.5 × 10^8^ colony-forming units (CFU)/mL, was used to standardize microbial inocula in order to guarantee consistency and reproducibility across all test settings. This standard is prepared by mixing 1% sulfuric acid (H_2_SO_4_) with 1% barium chloride (BaCl_2_) to produce a turbid solution with a known optical density at 600 nm. A visible spectrophotometer was used to adjust the turbidity of each test organism suspension to match this standard. This technique eliminated the variability caused by inoculum size by ensuring that the starting concentration of all microorganisms was the same across test groups and pH conditions.

### 4.7. Antimicrobial Activity Testing

Using the broth dilution method, the antimicrobial efficacy of each titanium surface treatment was assessed under different salivary pH conditions. This method is a common in vitro approach used to evaluate the growth of bacteria and fungi in liquid culture media. Each of the titanium specimens was aseptically transferred into a sterile glass beaker containing 10 mL of nutritional broth that was inoculated with a standardized microbiological suspension (using the 0.5 McFarland standard). Using the corresponding buffer solutions previously mentioned, the broth was first adjusted to one of the three test pH levels (4.5, 6.5, or 8). All samples were incubated for 24 h at 37 °C under aerobic conditions, enabling considerable time for microbial interaction with the surface coatings.

Using a visible spectrophotometer set at 600 nm, the optical density (OD) of broth culture was assessed following incubation. Microbial growth was proxied by the solution′s turbidity; higher turbidity denoted increased microbial proliferation, whereas lower OD values indicated growth inhibition.

## 5. Conclusions

This in vitro study demonstrates that CeHAp and BiHAp coatings on CpTi exhibit **distinct, pH-sensitive antimicrobial effects**, with CeHAp showing a **superior inhibition of *S. aureus*** at neutral pH—conditions relevant to early implant healing. While antimicrobial effects were less consistent for *E. coli* and *C. albicans*, CeHAp maintained broader efficacy across pH ranges. These findings underscore the potential of CeHAp coatings to limit early microbial colonization under physiologic conditions. Future work should target **anaerobic pathogens** and explore **host tissue responses** under dynamic intraoral pH to support clinical translation.

## Figures and Tables

**Table 1:** Analysis of microbial growth of *S. aureus*, *E. coli*, and *C. albicans* at pH 4.5 using Kruskal–Wallis ANOVA test.

Parameters	GROUPS	N	Median	IQR	$\chi^{2}$	*p* Value
***S. aureus* at pH 4.5**	CpTi	3	0.1	0.05	2.44	0.295
	BiHAp	3	0.003	0.01		
	CeHAp	3	0.05	0.015		
***E. coli* at pH 4.5**	CpTi	3	0.11	0.4	5.60	0.061
	BiHAp	3	0.05	0.01		
	CeHAp	3	0.18	0.025		
***C. albicans* at pH 4.5**	CpTi	3	0.14	0.03	5.60	0.061
	BiHAp	3	0.04	0.015		
	CeHAp	3	0.13	0.02		

**Table 2:** Analysis of microbial growth of *S. aureus*, *E. coli*, and *C. albicans* at pH 6.5 using Kruskal–Wallis ANOVA test.

Parameters	GROUPS	N	Median	IQR	χ ^2^	*p* Value
***S. aureus* at pH 6.5**	CpTi	3	0.24	0.01	7.38	0.025
	BiHAp	3	0.01	0.005		
	CeHAp	3	0.03	0.005		
***E. coli* at pH 6.5**	CpTi	3	0.27	0.02	7.26	0.027
	BiHAp	3	0.07	0.01		
	CeHAp	3	0.05	0.01		
***C. albicans* at pH 6.5**	CpTi	3	0.16	0.03	2.62	0.270
	BiHAp	3	0.1	0.1		
	CeHAp	3	0.1	0.015		

**Table 3:** Analysis of microbial growth of *S. aureus* at pH 6.5 using Dwass–Steel–Critchlow–Fligner pairwise comparison.

Pairwise Comparison of *S. aureus* at pH 6.5	GROUPS	W	*p* Value
**CpTi vs. BiHAp**	CpTi	−0.953	0.779
**CpTi vs. CeHAp**	BiHAp	−0.939	0.785
**BiHAp vs. CeHAp**	CeHAp	2.818	0.114

**Table 4:** Intergroup analysis of microbial growth of *E. coli* at pH 6.5 using Dwass–Steel–Critchlow–Fligner pairwise comparison.

Pairwise Comparison of *E. coli* at pH 6.5	GROUPS	W	*p* Value
**CpTi vs. BiHAp**	CpTi	−2.78	0.121
**CpTi vs. CeHAp**	BiHAp	−2.82	0.114
**BiHAp vs. CeHAp**	CeHAp	−2.82	0.114

**Table 5:** Analysis of microbial growth of *S. aureus*, *E. coli*, and *C. albicans* at pH 8 using Kruskal–Wallis ANOVA test.

Parameters	GROUPS	N	Median	IQR	χ ^2^	*p* Value
***S. aureus* at pH 8**	CpTi	3	0.2	0.01	6.83	0.033
	BiHAp	3	0.12	0.005		
	CeHAp	3	0.11	0.00		
***E. coli* at pH 8**	CpTi	3	0.1	0.01	6.25	0.044
	BiHAp	3	0.15	0.01		
	CeHAp	3	0.13	0.015		
***C. albicans* at pH 8**	CpTi	3	0.15	0.015	6.06	0.048
	BiHAp	3	0.16	0.015		
	CeHAp	3	0.2	0.01		

**Table 6:** Intergroup analysis of microbial growth of *S. aureus* at pH 8 using Dwass–Steel–Critchlow–Fligner pairwise comparison.

Pairwise Comparison of *S. aureus* at pH 8	GROUPS	W	*p* Value
**CpTi vs. BiHAp**	CpTi	−2.82	0.114
**CpTi vs. CeHAp**	BiHAp	−2.95	0.093
**BiHAp vs. CeHAp**	CeHAp	−2.24	0.254

**Table 7:** Intergroup analysis of microbial growth of *E. coli* at pH 8 using Dwass–Steel–Critchlow–Fligner pairwise comparison.

Pairwise Comparison of *E. coli* at pH 8	GROUPS	W	*p* Value
**CpTi vs. BiHAp**	CpTi	2.78	0.121
**CpTi vs. CeHAp**	BiHAp	2.78	0.121
**BiHAp vs. CeHAp**	CeHAp	−1.88	0.379

**Table 8:** Intergroup analysis of microbial growth of *C. albicans* at pH 8 using Dwass–Steel–Critchlow–Fligner pairwise comparison.

Pairwise Comparison of *C. albicans* at pH 8	GROUPS	W	*p* Value
**CpTi vs. BiHAp**	CpTi	1.59	0.5
**CpTi vs. CeHAp**	BiHAp	2.78	0.121
**BiHAp vs. CeHAp**	CeHAp	2.78	0.121

## Data Availability

The data that support the findings of this study are available from the corresponding author upon reasonable request.
